# Early experiences on the feasibility, acceptability, and use of malaria rapid diagnostic tests at peripheral health centres in Uganda-insights into some barriers and facilitators

**DOI:** 10.1186/1748-5908-7-5

**Published:** 2012-01-23

**Authors:** Caroline Asiimwe, Daniel J Kyabayinze, Zephaniah Kyalisiima, Jane Nabakooza, Moses Bajabaite, Helen Counihan, James K Tibenderana

**Affiliations:** 1 Malaria Consortium Africa Regional Office, Plot 25 Upper Naguru East Road, Kampala, Uganda; 2 Foundation for Innovative New Diagnostics, Plot 23A Akii Bua Road, Kampala, Uganda; 3 Women and Gender Studies Department, Faculty of Social Sciences, Makerere University, Kampala, Uganda; 4 National Malaria Control Programme, Ministry of Health, Loudel Road, Kampala, Uganda; 5 Bugembe Health Centre IV, Jinja, Uganda; 6 Malaria Consortium, Development House, 56-64 Leonard Street, London, UK; 7 Disease Control and Vector Biology Unit, London School of Hygiene and Tropical Medicine, Keppel Street, UK

## Abstract

**Background:**

While feasibility of new health technologies in well-resourced healthcare settings is extensively documented, it is largely unknown in low-resourced settings. Uganda's decision to deploy and scale up malaria rapid diagnostic tests (mRDTs) in public health facilities and at the community level provides a useful entry point for documenting field experience, acceptance, and predictive variables for technology acceptance and use. These findings are important in informing implementation of new health technologies, plans, and budgets in low-resourced national disease control programmes.

**Methods:**

A cross-sectional qualitative descriptive study at 21 health centres in Uganda was undertaken in 2007 to elucidate the barriers and facilitators in the introduction of mRDTs as a new diagnostic technology at lower-level health facilities. Pre-tested interview questionnaires were administered through pre-structured patient exit interviews and semi-structured health worker interviews to gain an understanding of the response to this implementation. A conceptual framework on technology acceptance and use was adapted for this study and used to prepare the questionnaires. Thematic analysis was used to generate themes from the data.

**Results:**

A total of 52 of 57 health workers (92%) reported a belief that a positive mRDT result was true, although only 41 of 57 (64%) believed that treatment with anti-malarials was justified for every positive mRDT case. Of the same health workers, only 49% believed that a negative mRDT result was truly negative. Factors linked to these findings were related to mRDT acceptance and use, including the design and characteristics of the device, availability and quality of mRDT ancillary supplies, health worker capacity to investigate febrile cases testing negative with the device and provide appropriate treatment, availability of effective malaria treatments, reliability of the health commodity supply chain, existing national policy recommendations, individual health worker dynamism, and vitality of supervision.

**Conclusions:**

mRDTs were found to be acceptable to and used by the target users, provided clear policy guidelines exist, ancillary tools are easy to use and health supplies beyond the diagnostic tools are met. Based on our results, health workers' needs for comprehensive case management should be met, and specific guidance for managing febrile patients with negative test outcomes should be provided alongside the new health technology. The extent, to which the implementation process of mRDT-led, parasite-based diagnosis accommodates end user beliefs, attitudes, perceptions, and satisfaction, as well as technology learnability and suitability, influences the level of acceptance and use of mRDTs. The effectiveness of the health system in providing the enabling environment and the integration of the diagnostic tool into routine service delivery is critical.

## Background

The benefits of malaria rapid diagnostic test (mRDT) technologies are well documented [1,2] and globally acknowledged. World Health Organisation (WHO) has endorsed mRDTs as adjunct tests to microscopy for parasitological confirmation of malaria in routine fever case management at lower levels of healthcare [2]. However, endorsing a new or improved health technology in itself does not guarantee end-user utilisation, especially in resource-poor countries where government health facilities are underfunded, ineffective, or underutilised [3]. Studies done in settings with well-resourced health services have reported several factors responsible for acceptance and use of new digital and health technologies [4,5]. These factors include organisational features such as how well the new technology is integrated with existing technologies, workflow, and top management commitment to the new technology. Also cited are individual factors such as perceptions of negative effects on users, resistance to change, lack of control, and readiness for change, as well as job factors such as self-efficacy, level of education, previous experience with similar technology, age, gender, clarity on the reasons for the new technology, training, and participation. Other factors reported include the design of guidelines or models of implementation that accommodate a range of end-user expectations. There are some discussions that successful introduction and uptake should be informed by credible field experiences and predictive variables for technology assimilation and acceptance [6,7]. It is not clear whether similar factors are responsible for assimilation of new technologies such as mRDTs in low-resource settings.

In most parts of Africa where malaria-like fevers are responsible for more than 300 million episodes and one million deaths per year [1,8], parasite-based diagnosis with low-cost, simple diagnostic tools is recommended to confirm or rule out malaria. These technologies, if deployed and used optimally, should enable targeted treatment of malaria at lower-level healthcare facilities and at the community level [2]. In 2006, the National Malaria Control Programme (NMCP) of the Ministry of Health of Uganda requested local research groups for evidence to inform a shift from presumptive to targeted treatment of malaria using mRDT-led parasitological diagnosis at peripheral and community levels of healthcare. At the time, microscopy was the recommended form of parasite-based diagnosis at facilities with the necessary equipment and personnel, *i.e*., hospitals, large health centres grade IV (HC IV) and some mid-level health centres grade III (HC III). Clinical diagnosis was recommended for peripheral health centres grade II (HC II) and presumptive treatment at the community level by community-based agents (referred to at the time as community medicine distributors).

NMCP and its stakeholders reviewed the international and national evidence that was available at the time and agreed via consensus to go for a phased approach in deploying mRDTs to a national scale, and in a manner that complemented microscopy-based diagnosis [9]. This study and other operational research [10] were conceived and carried out to facilitate evidence-based policy formulation and high quality implementation of mRDT-led, parasite-based diagnosis.

This study comprised qualitative and quantitative descriptive work. The effect of mRDTs on antimalarial drug prescription practices was assessed using data extracted from routine health management information records (HMIS) at health facilities. The findings of this quantitative part of the study have been reported elsewhere [11]. The qualitative aspects reported here sought first-hand information on the early experiences of health centre attendees' and health workers with mRDTs. Specifically, perceptions, attitudes, beliefs, and practices of these users were documented and assessed to gain insight on the barriers and facilitators of acceptance and use of mRDTs.

## Methods

This work involved research embedded [12] into the implementation process of mRDT-led, parasite-based diagnosis. The study took place between July and December 2007, whereas the longer implementation process started in early 2006. Data were collected using two sets of surveys targeting health workers and health centre attendees. A conceptual framework that was adopted from previous work on technology acceptance [13] was used to inform the design of the questionnaires and analysis of the data.

### The implementation team

The study implementation team consisted of a clinical epidemiologist, a parasitologist, and a laboratory technologist, who provided study oversight, trained health workers, and provided support supervision. Ten male and female research assistants with backgrounds in clinical and social sciences were selected and trained to carry out the interviews. In the choice of research assistants, some experience in qualitative research and fluency in the dialects of interviewees were taken into consideration. All team members participated in the pre-testing of the study instruments.

### Study design

This was a qualitative descriptive design. Data were collected from a sample of health workers and health centre attendees in five purposively selected districts, namely Kapchorwa, Mubende, Iganga, Jinja, and Mbale. Based on the objectives of the larger study, districts were selected to represent the malaria transmission and rural-urban settings found in the country. In this regard, Kapchorwa represented a hypo-endemic area with a malaria parasite prevalence of < 20%; Mubende a mesoendemic area with a malaria parasite prevalence of 20 to 70%; Iganga a hyper-endemic region with malaria parasite prevalence of > 70% [14]; Jinja and Mbale were included to represent a population located in relatively semi-urbanised areas compared to the other districts that were relatively more rural. These stratifications were considered important to better contextualise the findings, but have not been used in this paper.

For inclusion, HCs had to be part of the public healthcare sector, not have serological and parasite-based diagnostic services, and with no previous involvement in medical or operational research. When contacted, the HC in-charges had to express willingness to participate in the study. With these criteria, five HCs per district, of types II and III (the average total number of public HCII and III in our study area was 21 in Kapchorwa, 49 in Iganga, 48 in Jinja, 35 in Mubende, 14 in Mbale) were randomly selected by the district health officers within each of the five districts for the study. Of these, one HC per district was randomly allocated to be a comparator HC without mRDT-led, parasite-based diagnosis. However, one comparator HC in Iganga district was converted to an implementation HC, after mRDTs were introduced in August 2007 by another partner organisation operating in the district. To replace this, another HC was purposively selected by the malaria focal person in the district to be the comparator. In this way, 21 health centres where mRDTs were deployed formed the study sample for this component of the research.

The implementation process involved mRDT selection and deployment, community sensitisation, and health worker training and supervision. These steps are described below.

### mRDT selection

Given the predominance of *P. falciparum *as the cause of malaria in this setting, it was decided to use a histidine rich protein-2 (HRP2) type of mRDT. In deciding the mRDT brand to use, a basic assessment of ease-of-use was carried out on four brands amongst nine health workers at a health centre not involved in this study. The ICT Pf brand was chosen on the basis of packaging and labelling, ease of performance, readability of the results, cost, heat stability data, and reported sensitivity and specificity. The accuracy of the ICT Pf brand had earlier been established in Uganda [15], and the study findings were used to inform training as well as boost health workers' confidence in the mRDT.

### mRDT deployment

mRDTs were quantified, procured, handled, and stored safely by Malaria Consortium before delivery to implementing HCs in Mbale, Kapchorwa, and Mubende districts in June 2007. Job aids and ancillary supplies such as cotton wool, timers (wall clocks), indelible markers for labelling, sharps containers, and disposal bins were provided alongside mRDTs. Iganga and Jinja districts mRDTs received mRDTs supplies without gloves, clocks, sharps boxes, and indelible markers two weeks after the initial districts. In-charge health workers in the latter HCs were encouraged to order these supplies through the routine district medical supplies system. Their efforts to do so were not successful, and as a result these additional items were supplied about two weeks later. The delay in distributing these items to these areas provided the opportunity to observe what effect the provision or lack of mRDT ancillary supplies had on health workers' decisions on whether or not to use mRDTs.

### Community sensitisation

Prior to health worker training, the district health teams and local public opinion leaders were informed about this study. Opinion leaders were given the chance to discuss issues related to the research. The purpose of this process was to garner their support for the work and make them aware of the benefits of better malaria diagnosis in fever case management.

### Training of health workers

Guided by an earlier version of the WHO generic mRDT trainers' manual [16], a one-day, hospital-based training for district officials and health workers was conducted at larger HC IV facilities in the participating districts. Trainers, comprised of three study team supervisors and one technical member of the National Malaria Control Programme, taught HWs how to perform, interpret, and utilise mRDTs in fever case management. A script concordance test (SCT) [17] was used to evaluate the degree of concordance between health workers proficiency in performing a mRDT and the pictorial job aid with step-by-step instructions. At the end of the training session, each health worker was observed performing three mRDTs and an average SCT score calculated. *A priori*, the health workers were not informed that a SCT on their practice would take place. However, health workers were informed about the interviews as part of the upcoming data collection process. It was only after trainers had observed trainees performing the tests and interpreting the results that coaching was given to mitigate any errors. A score of 95% (one out of 15 steps skipped or poorly performed) and above reflected a high degree of concordance corresponding to optimal mRDT use. This was set as the minimum tolerable performance on an average of three tests performed per health worker.

### Health worker supervision

For the first eight weeks, supervision occurred at two-week intervals. Subsequently, supervision took place once a month. A pre-designed mRDT supervision checklist was used alongside the national routine technical supervision tool, which did not have a section on mRDTs at the time. During supervisory visits, health workers in implementation HCs were encouraged to use the diagnostic tool and test results in fever case management. During each supervision visit, the SCT was used to measure the degree of concordance of the health workers' knowledge of mRDT job aid instructions and the efficiency with which this knowledge was translated into clinical actions. Supervision visits were also used for data collection.

### Conceptual framework

**Figure 1:**
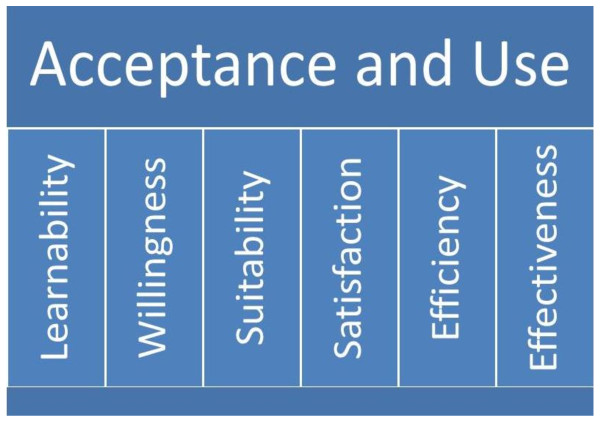
**Conceptual framework for exploring acceptance and use of malaria rapid diagnostic tests when introduced in public sector lower level health centers in Uganda, adapted from Jeng J (2004)**.

To guide the research, a conceptual framework was adopted based on earlier models [4,13,18] and publications [19]. The framework is depicted in Figure 1. In the framework, feasibility is defined as the process in which mRDTs are deployed to HCs, leading to their acceptance and utilisation by end users. The framework also recognises that feasibility depends on acceptance and use factors as well as a host of implementation factors, such as policy, case management guidelines, supplies, budgeting, planning, monitoring, and evaluation.

As illustrated in Figure 1, it can be presumed that mRDT acceptance and use are potentially influenced by attributes related to users, *i.e*., health workers and HC attendees, as well as the diagnostic tool and the health system. These attributes, including learnability, willingness, suitability, satisfaction, efficacy, and effectiveness have been identified in other settings [18]. For this study these attributes were adapted with the meanings below;

1. Learnability: ability of the health worker to understand how to correctly perform the mRDT, a new health technology, and accurately read the test results.

2. Willingness: health worker intention to carry out a blood test each time it is necessary, wait for the results, and prescribe medication (or not) in line with national guidance and test results. Regarding the HC attendee, willingness was defined as HC attendees' intention to have the test performed on themselves or their child, wait for test results, and take medication (or not) in line with the test results.

3. Suitability: health workers' belief that the test is relevant for his/her work and that test results are a true indication of the presence or absence of malaria parasites. Regarding HC attendees, suitability was defined as HC attendees' belief that the test is relevant in determining whether or not they or their child has malaria.

4. Satisfaction: a health worker's feeling that the test is convenient to perform and that it is a process he/she likes doing. Regarding the HC attendee, suitability was described as feeling that a test is convenient to take and that it is a process they would like to carry out again. It also refers to the ease-of-use of the mRDT, which is affected by the design of the mRDT, its labelling, and instructions.

5. Efficacy: that the health worker is able to make the effort and time to perform a test, read, interpret, and record test results, as well as prescribe medication in line with the test results, as part of their daily routine work.

6. Effectiveness: that the enabling organisational and supporting systems, such as training, supervision, job aids, supplies, medicines, space, lighting, timers, storage, and disposal are present or carried out and are integrated into existing routine systems.

These attributes work in an interrelated way to contribute to acceptance and use of a new technology. Acceptance comprises positive perceptions, beliefs, and attitudes toward mRDTs and test results among users, i.e., health workers and health centre attendees. Use refers to the actions taken by health workers to apply the tool and its results to achieve specified outcomes. In turn, if acceptance and use are high, then implementation is feasible. This conceptual understanding informed more focused inquiries in the design of the data collection tools.

### Implementation and data collection tools

A WHO training manual and mRDT job aid that were undergoing review at the time, as well as a modified NMCP supervision checklist and a SCT were used. A health worker semi-structured interview guide and a patient exit (*i.e*., HC attendee) interview guide were used for one-to-one interviews administered by research assistants. The interview guide comprised closed and open-ended questions that focussed on the study objectives in such a way that these data could be used to generate information for the thematic analysis informed by the conceptual framework. All data collection tools were pretested at Kasangati HC IV, a health facility not participating in the study.

### Ethical considerations

This research was approved by the NMCP, Ministry of Health of Uganda, and the Uganda National Council of Science and Technology (UNCST). The procedures followed were in accordance with the Helsinki Declaration. In addition to asking the HC in-charge for oral consent for that HC to be included in the study, each health worker was asked to consent by signing the consent form to participate in the study. The HC attendees' consent was obtained by a research assistant before the exit interview. The consenting process for both health workers and HC attendees included an explanation of the study, its objectives, potential benefits and risks, and the contact information of the study PI. The HC attendees gave a witnessed signature or thumb-printed approval to participate.

### Interview procedures

#### Health worker interviews

Following mRDT deployment, interviews were conducted on a monthly basis using semi-structured interview guides designed to get a broad insight into mRDTs and parasite-based diagnosis. Open-ended interview questions were administered by study clinical and social work research assistants. The first of four rounds of interviews were conducted with health workers who had given formal consent, six to eight weeks after the initial deployment of mRDTs in June 2007. Interviews were conducted at the study HCs during working hours and in a manner that avoided disruptions to service delivery. All eligible health workers had a minimum one-month experience with mRDTs and were involved in fever case management.

#### Health centre attendee interviews

The study included all HC attendees who agreed to be interviewed exiting the study HC after receiving care from the health workers. At the point of exit, the attending health worker told the patient about the visiting study team, and that they were interested in talking to the patient or parent/guardian of the patient younger than 18 years. Those HC attendees who were 14 to 18 years and visiting the health centre on their own, such as teenage pregnant girls and mothers, were consented as unique cases, referred to as emancipated and mature minors by UNCST. Although the health workers in the HC knew that the HC attendees were being asked questions related to mRDTs, they were not aware of the questions in the HC attendee questionnaire. HC attendees were informed by the research assistants about the study, the length and format of the interview, terms of confidentiality, and the right to withdraw consent before or during the interview. A semi-structured questionnaire and photographic aids of mRDTs and the first line antimalarial medicine, artemether-lumefantrine (AL), were used to simplify explanations during interviews. The interview focused on: knowledge and perceptions of malaria infection; parasite-based diagnosis and treatment; willingness to test and re-test with mRDTs; belief in the test result; and the HWs decisions on healthcare, following a blood test for malaria. Discussions with interviewees were guided by questions seeking to understand opinions and beliefs about mRDT-led, parasite-based diagnosis, as well as barriers and facilitators of mRDTs acceptability.

### Quality Assurance

The mRDTs were transported in vehicles that allowed free flow of air. Manufacturer's temperature specifications (4 to 30°C) were monitored and maintained both at storage and during transportation using log tags. To enhance clarity, indelible markers were provided to label the mRDT cassette with patient identifiers, date, and time when to read the test results. All health workers retained used mRDT cassettes in the HC during the study period, because guidance on appropriate disposal methods was anticipated from national level. Study research assistants were trained to carry out interviews and were regularly overseen by three study supervisors throughout the data collection period. All completed interview questionnaires were checked for accuracy and completeness at the end of each month during a health facility visit by the study statistician.

### Data analysis

Thematic analysis using a realist method was used to generate themes on the acceptance and use of mRDTs among respondents. This process started with the manual transcription of all the qualitative data from completed interview questionnaires. This step was largely carried out by the social scientist; the research team reviewed the transcriptions and through a number of group discussions identified meaningful patterns in the data from ideas, views, opinions, perceptions, and beliefs of respondents. These patterns were annotated with numerical codes. They were reviewed and assessed in line with the attributes of the conceptual framework to form themes that related to acceptance and use. The implications of these themes on implementation feasibility were used to categorise them into barriers and facilitators. With regard to the quantitative data, which relate more to mRDT use, the effect of mRDTs on anti-malarial drug (AMD) prescription was quantified by computing risk ratios for the two analysis designs (pre-post and intervention-control), after adjusting for clustering in health facilities using survey data analysis methods in STATA 10. This quantitative information is presented elsewhere [11].

## Results

As part of the implementation process, 129 health workers, of 135 who were eligible, were trained to perform mRDTs and utilise test results in fever case management. Of those who completed training, 74 were clinical officers, six were laboratory technicians, seven were records assistants, and 42 were general service support staff such as health educators, nursing assistants, and vaccinators. Six health workers that had missed the initial training session received on-the-job training during the first supervisory visit, one month (*i.e*., in August) after mRDT deployment.

A total of 102 health workers (76% of all eligible health workers trained centrally and on site) consented to be interviewed. The remaining 33 health workers were not available at their stations at the time of the interview for various reasons. During the study period, complete interviews were carried out with 63 of 102 (62%) health workers because some of those who gave their consent were not able to take part in the interviews due to a number of reasons. The main reasons for non-participation after consenting were that the health worker had to attend to patients who arrived before or during the interview and some health workers felt that one month of mRDT implementation was not enough for them to have sufficient experience with mRDTs to answer the questions.

A total of 1,068 patients (829 adult patients and parents/guardians of 239 children age five years or younger) were interviewed at exit following care at health centres where mRDT-led, parasite-based diagnosis was introduced. The majority of HC attendees interviewed, 65%, were female (697 mothers and 70 guardians). The findings presented here are limited to the 97% (1,035/1,068) of the HC attendees who actually completed the interviews. The key findings are presented here as themes and discussed. Acceptance and use barriers and facilitators are illustrated at end.

### Health Worker experiences related to mRDTs deployment

#### Adherence to the mRDT job aid

During the pre-deployment training session, health workers generally perceived the mRDT job aid as a useful tool. A total of 54% (70/129) of health workers correctly carried out all 15 steps in the mRDT job aid, or missed only one. Some health workers skipped more than one instruction or performed the mRDT without the job aid. SCT findings indicated that the instruction that proved to be most difficult was pipetting blood using the Pf mRDT ICT™ blood transfer device. Only 18/63 health workers drew blood accurately during the first supervision, three weeks after deployment. Other common errors included using an incorrect number of drops of running buffer (43/63 health workers), forgetting to clean the finger with alcohol swab before making the finger prick (47/63 health workers), reading the test results at an incorrect time (45/63 health workers) or forgetting to check the expiry date on the test kit. Although similar remarks such as, 'RDTs are good if you are still young. With old age, you shake and you cannot do it. It is also hard to tell the mark of the test and control lines if it is not bright outside' were made by some elderly health workers in Jinja and Kapchorwa, by the second round of supervision, six weeks later, 32 (51%) health workers who found it hard to use the blood collecting device had become familiar with it.

#### Characteristics of the mRDT kit

The ICT Pf. blood collection device proved difficult to use mainly because of the skill needed to pipette 5 μl of blood accurately. Interpreting the mRDT expiry date on the package seemed to be a common challenge at all health centres. It was not clear to them because the date indicated only the month and year as 12/2007. Some health workers interpreted this as expiring on the first day of the month whereas others decided that the mRDT expired on the last day of the month. A male laboratory staff member at a HC III, Mbale District, reasoned that, 'You know the antigen in the RDT is a protein, which is unstable in our conditions. I think it is better not to stretch the expiry date further than the first date of the month.'

Some health workers perceived a faint mRDT test line as signifying less malaria infection than a bold test line. These health workers reported that children were more likely to have bold test lines than adult patients. A male health worker at HC III, Kapchorwa District explained that, 'we have noted that adult patients have a faint positive test line unlike children, so we give the old people CQ [chloroquine] and the young ones get Coartem [AL] yellow. Otherwise we would have to combine the Coartem [AL] yellow for the adults and it would run out quickly.'

Some of the laboratory technicians expressed concern that mRDTs are not able to quantify malaria parasites and that very low density antigenemia could be undetected by mRDTs and lead to severe forms of malaria in patients with false negative results. A laboratory assistant at a HC III in Mbale said that, 'RDTs cannot tell you the severity of malaria, so we just take precaution and treat or I send the patient for microscopy.'

#### Satisfaction with the mRDT

Two months after introducing the new health technology to the health centres, 57 (90%) health workers reported enthusiasm to use mRDTs on a daily basis and felt that mRDTs were relevant tools for fever case management. It emerged that the health workers at 12 of 21 HCs felt that health centre attendees had more confidence and respect in them because of their capacity to perform the test. For example, an in-charge from a HC III in Mubende district reported that, 'the community now has confidence in us and the services we offer because of the RDTs.'

Another in-charge from a HC III, in Mbale district said, 'with blood tests, we can now confidently tell that the patient is not suffering from malaria.'

One other perceived benefit of the diagnostic tool was that it allowed the health workers to monitor the effect of malaria interventions such as distribution of long-lasting insecticide treated nets (LLINs) and the use of indoor residual spraying (IRS). An in-charge in Mubende District mentioned that, 'with RDTs, we can now see the impact of spraying mosquitoes and distributing bed nets. Malaria cases are very low.'

### Work and organisational environment

The small size and nature of the alcohol swabs supplied with the mRDT kit seemed to pose a challenge because one swab was not sufficient to clean soiled hands, which can be common in rural agricultural settings. Without adequate clean tap water at the HCs, health workers were often faced with a situation in which they are expected to perform mRDTs on patients presenting with soiled fingers without the means to clean them properly. In this study, we provided cotton wool to supplement the small and thin alcohol swabs supplied with the mRDT. It was common for health workers not to have a wristwatch or wall clock to time the testing process, and yet they were trained to use a timer for the process.

There was the perception that mRDTs placed additional pressure on health workers due to community demand for the new diagnostic tool. A HC III in Iganga district, which was manned by four health workers decided to charge a fee (equivalent to about $0.20) for each mRDT performed as a way of controlling patient demand. The in-charge confirmed this, after a female health centre attendee inquired during an exit interview, 'Musawo (health worker) told us that in order to have a malaria blood test, we have to pay 500 shillings, which help him to bring in more tests. Is this true?'

Noteworthy, 74% (47/63) of all health workers thought it wasteful to change gloves from one patient to the next. Overall, 57% (36/63) reported failure to regularly perform the test due to programmatic constraints, such as lack of ancillary supplies, heavy workload, inadequate staffing, and unclear national guidelines.

### Treatment practices in the context of mRDT results

Regarding choices to treat patients based on test results, only 57 of 74 of the clinical health workers accepted to address this question, and the other 20 were non-committal. About 75% (43/57) reported consistent utilisation of the mRDT results when managing patients with suspected malaria infection. A total of 16 of 57 (28%) reported that they would not necessarily treat every patient testing positive with anti-malarials. A total of 23 of 57 (40%) mentioned that they do not rule out prescribing antimalarial medicine to a patient testing negative for malaria infection with a mRDT. Some health workers said that they prescribed non-ACT malaria treatment to HC attendees above five years of age with a positive mRDT result in order to preserve ACTs for patients aged below five years. The non-ACTs prescribed as first line treatment for malaria were quinine, CQ, or sulphadoxine-pyrimethamine.

The proportion of health workers that did not prescribe ACT or non-ACT anti-malarials to patients testing negative or 'slightly' positive, gave folic acid, multivitamins, or analgesics. This category of health workers reasoned that they were saving ACT for those who were mRDT positive and still meeting the expectations of patients who insisted on getting a treatment for their complaints.

A female nurse at HC III, Mubende district said, 'in case of pending stock outs, negative RDT adult patients and slightly positive RDT patients get CQ/SP, in order to save Coartem for children.'

A male in-charge at a HC III in Mubende District articulated that, 'children get Coartem [AL] even if they test negative, according to policy.'

Another one from a HC III in Mubende said, *'*my judgement as a clinician guides my treatment decision when the RDT is negative. Patients expect us to treat them regardless of RDT result.

A female nurse at HC III, in Mubende district reported that, 'we prescribe multivitamins, paracetamol or folic acid to adult patients with negative RDT results. In case of pending stock outs for Coartem, negative RDT adult patients and slightly positive RDT patients get CQ/SP, in order to save Coartem for children.'

### HC attendee experiences related to mRDT deployment

#### Willingness to have a test

More than 94% (977/1035) of HC attendees who completed the interviews mentioned willingness to have a mRDT performed on them or their children. About 3% of health centre attendees interviewed (36 respondents) were reluctant to take the test despite having never experienced the procedure before. Some respondents (59%) who were willing to take a blood test believed that they were justified to challenge or reject a negative mRDT result, if it was not associated with a drug prescription. The expectation of an antimalarial drug prescription despite a negative mRDT result was noted among some respondents in all implementation health centres. This was best expressed by a male health centre attendee at a HC III, Mubende District who said, 'I like the idea of taking a blood test, but I still need to get treated even if the test says I have no malaria. Would I have come to the clinic if I was healthy?'

Other reasons influencing willingness to accept a mRDT were related to gender, spiritual, and traditional beliefs. A mother with child at HC II, Jinja District said, 'I need my husband to allow me to give the child's blood for testing.' A family head attending with family at HC III, Mubende District said, 'my religion does not permit us to give blood.' And an elderly woman attending a HC II, Jinja District remarked that, 'I cannot tell where my blood will end up, since it is placed in a container and retained.'

#### Willingness to wait for the test result

Asked if attendees were willing to spend longer than the usual waiting time at the HC as a result of the mRDT procedure, 99% (of those willing to take the blood test) said they were willing to wait for the mRDT result if they had to. Lack of confidence in the mRDT result, dissatisfaction with the decision of the health worker not to give malaria treatment, or fear of the pain of the finger prick were the main reasons for reluctance to have a test done. Some patients considered testing as a waste of time, or perceived the test results as false, preferring to believe that malaria was the cause of the febrile illness.

## Discussion

This study provides the first documentation of implementation feasibility and in-depth account of acceptance and use of mRDTs and test results in fever case management at lower level health facilities in Uganda. It provides a rich source of information that is of benefit to policy makers and implementers because it was carried out in typical programmatic settings and in different geographical locations. The results demonstrate the multifactorial nature of introducing new health technologies in resource-limited settings and highlight some beliefs, perceptions, and reasons for adhering or disregarding test results in malaria case management. To some extent, these findings are similar to other reports in well-resourced settings, in that acceptance and use of a new digital or health technology depends on a host of implementation issues, as well as individual, cultural, and organisational factors [4,13,18,20]. The findings from this study suggest that the introduction and scaling up of new health technologies in Uganda require a specific policy framework and that implementation models address more than the common issues of health worker training, commodity distribution, and support supervision.

The conceptual framework that was adopted to inform our inquiries helped to elucidate the broader contextual issues. These considerations include: the design and characteristics of the mRDT (which contribute to learnability and suitability); the availability and quality of ancillary supplies for performing the mRDT (which contribute to effectiveness); health worker capacity to investigate mRDT negative febrile cases and provide appropriate treatment (efficacy and effectiveness); availability of effective malaria treatments (effectiveness); reliability of the health commodity supply chain (effectiveness); and dissemination of policy recommendations (effectiveness); individual dynamism (efficacy); vitality of supervision and feedback (effectiveness); and strategic approaches that support integration of new tools into existing systems without jeopardising health worker's perceived sense of respect (satisfaction and efficacy) among health centre attendees.

The findings that health workers perceived mRDTs as a symbol of professionalism in healthcare and a majority of HC attendees associated the mRDT with quality care by the health worker who performed the mRDT are potentially a significant indication of the perceived role of this new point-of-care health technology.

Some health workers felt the mRDT was an empowering tool that enabled them to engage patients in the decision making regarding their treatment. This probably implied a sense of confidence to both the health worker and HC attendee when compared to the process of clinical diagnosis or presumptive treatment that mainly relies on the health worker's judgement. The relevance of patient involvement in decision making and health workers interpersonal skills in enhancing patient willingness to test has been recognised before [4,6]. It is likely that if prestige and patient confidence are associated with a new health technology such as the mRDT, acceptability is more likely to occur.

On the other hand, some health workers reported the restrictive nature of test results in clinical judgement, based on their perceptions that the new diagnostic tool imposed on them treatment decisions contrary what experience had taught them. The new tool seemed to pose a threat to health workers' capacity to make individual clinical judgments, which would in turn undermine health workers' credibility amongst their patients. This perception is noteworthy, particularly because the majority of the HCs attendees (98%) did indeed report that they had inquired about their test result. These facts suggest that the decision making process could no longer be monopolised by the health worker. One may want to consider the challenge of a new health technology that is perceived to undermine health workers' confidence and social status as a potential barrier to acceptance and use. This is a new diagnostic approach for health workers and their patients. It has been common practice over many years that the main cause of fever is malaria, and therefore clinical judgement and presumptive treatment have been the most appropriate way to handle fever cases.

Inconsistencies in policies and treatment guidelines were mentioned as a contributory factor for health workers' misconception of the role of mRDTs in fever case management. At the time of this study, the policy for malaria control and prevention in Uganda stated that, 'any patient with a history of fever within the last 24 hours without evidence of other diseases should be treated for malaria even with a negative blood smear for malaria parasites.... Given the current limitations of RDTs, their use should be considered only in special situations (epidemics, children under four months of age); and their routine use is therefore not recommended' [9].

Health workers' view on inconsistent policy guidelines is in concordance with earlier reports that associated ambiguous or lack of relevant health policies with poor use of malaria parasite-based diagnostic tools [21-24]. It is highly probable that regardless of the resource settings, supportive policies and guidelines play a major role in the acceptance of new health technologies and, in this case, use of test results.

Deploying the new diagnostic tool exposed some obstacles and opportunities resulting from inconsistencies in the pharmaceutical supply chain. The mRDT tool seemed to provide a practical way of targeting treatment and rationing ACT medicines for patients perceived to need them more with or without regard to the test result. In situations where ACT shortages were anticipated or present, health workers devised ways of preventing such shortages. For example, some health workers reported that some patients were given non-ACT malaria treatment CQ or sulfadoxine-pyrimethamine (SP), while others received folic acid, multivitamins, or analgesics. In this way, the health workers felt they could save ACT for those who were mRDT positive, and yet meet the expectations of patients who insisted on getting a treatment for their complaints.

Some of the decisions taken were to prescribe non-recommended anti-malarials to those that were mRDT negative or to limit ACT to children aged less than five years. It is possible that some health workers felt the need to conform to patient expectations of a medical prescription. This means that optimal mRDT utilisation and adherence to test results can only be achieved if there is predictable and uninterruptible supply of the recommended treatments at all levels of healthcare.

The inability of mRDTs to quantify disease and predict the severity of disease may be a limitation worth addressing in health worker training and quality assurance programme planning. This limitation was mentioned by some health workers, particularly those with a background in laboratory-based diagnostic services. If a test resulted in a negative result, some health worker respondents (who did not believe that all mRDT negative results were truly negative) perceived that the host immunity of adult patients prevented the mRDT from detecting low levels of the parasite. The same health workers perceived that mRDTs could not detect other forms of *Plasmodium sp*., which were thought to exist in some epidemiological zones in Uganda. The misinterpretation of weak positive mRDT test lines as signifying less malaria was worrying; it may be related to the knowledge that malaria parasitaemia can be quantified using microscopy, and probably related to lack of understanding of the reason for varied test band colour intensities. These perceptions and beliefs, as well as concerns related to reliability of test results, emphasise the need for detailed information from manufacturers and a robust quality assurance system when new health technologies are introduced. Health workers' fears of potential risks associated with perceived 'low undetectable' parasite densities progressing to severe malaria should not be ignored in training aids, training sessions, and support supervision.

Although a one-day training period suggested by an earlier draft of the WHO mRDT training manual [16] may be deemed insufficient for comprehensive fever case management training, our trainees benefited from utilising hospital settings where there was opportunity for immediate expert feedback and practical sessions. Problem identification and solutions were possible in real time. Our observations indicate that performing mRDTs is relatively easy to learn, as earlier reported [11,25]. Supervision is required to coach health workers in correcting errors identified, based on recommended national guidance. However, this kind of supervision, *i.e*., on-the-job coaching, was not part of routine supervision. This challenge should be mitigated through comprehensive training of trainers and supervisors beyond technical subject matter and should include adult learning techniques.

Results obtained from interviews with HC attendees seem to suggest that accepting to use the new diagnostic tool technology is reliant on medical prescription after testing, regardless of the test outcome. This may be influenced by a perceived benefit of a treatment after a test or a tradition of associating health centres with medication. Accepting to take a mRDT and then leaving the HC without medicine, in case of a negative mRDT result, was difficult to comprehend and accept by the HC attendees. This reasoning is also probably a mindset arising from the standard of care over the years of presumptive treatment of fevers [21-24]. This expectation may take some time and effort to change.

**Figure 2:**
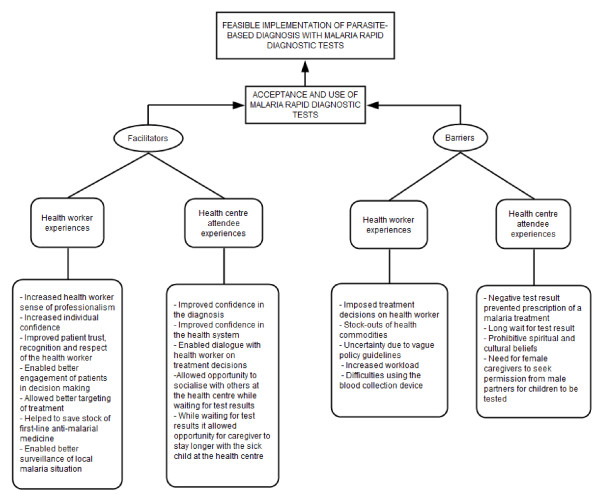
**Illustration of key facilitators and barriers of the acceptance and use of mRDTs in the early implementation of parasite-based diagnosis in public sector lower level health centres in Uganda**.

From a programmatic perspective, these findings seem to suggest other potential barriers and facilitators for successful implementation of mRDT-led, parasite-based diagnosis in resource-limited settings that may apply to other new health technologies; see Figure 2 for a summary of these. Although earlier reports [6,26,27] identified some factors affecting new health technology use in different settings, barriers and facilitators for accepting the new tool and test results were unknown in Uganda. In addition, this study identified similar factors for acceptance and use of new health technologies reported in well-resourced settings [5,6,27]. Our findings contribute to the body of evidence on new health technology acceptance and use, as well as factors determining optimal utilisation of test results in a resource-limited setting. The evidence provides an opportunity for further adaptation of a conceptual framework for mRDT acceptance and use in programmatic settings. The quality of introduction and implementation of other new health technologies can benefit from this and similar evidence.

One limitation of this study is that the findings are based on early experiences, which may not have allowed sufficient time for the respondents to form their opinions or make up their minds on the role of mRDT-led, parasite-based diagnosis. The effect of mRDTs on demand for diagnostic services and health facility attendance could not be ascertained during the study period with this design. Another limitation is that these data are of reported responses, which could mean that there is some degree of measurement error and social desirability bias. Despite these, the findings do contribute to other efforts seeking to inform successful introduction and adoption of new health technologies in resource-limited settings.

## Conclusions

This study provides an account of early experiences related to mRDT-led, parasite-based diagnosis among health workers and HC attendees at the frontline of the healthcare system. It was possible to illustrate the findings as barriers and facilitators for mRDT acceptance and use, which in turn affect feasible implementation of mRDT-led, parasite-based diagnosis. The findings indicate that mRDT acceptance and use is not only dependent on the perceived medical usefulness and availability of the tool, but also on the contribution that mRDTs make to intricate socio-cultural needs. These findings provide useful insights that can enhance the effectiveness of introducing new health technologies, such as mRDTs, at the level of peripheral health services in Uganda and other similar settings. Based on these early experiences, there are signs that mRDT-led, parasite-based diagnosis is feasible to implement with proper planning and careful consideration of a variety of factors that affect acceptance and use of this new diagnostic tool.

## Competing interests

The authors (CA, DK, ZK, JN, MB, HC, JKT) declare no competing interests.

## Authors' contributions

JT conceived the study and designed it with DK and CA. The entire team collaborated in the training and supervision of HWS. CA coordinated the implementation of the study, and ZK led data quality control and analysis with CA. CA and DK prepared the study report and CA wrote this manuscript with collegial reviews from the entire study team. All authors read and approved the final manuscript. All authors read and approved the final manuscript.
